# *DISC1* Ser704Cys impacts thalamic-prefrontal connectivity

**DOI:** 10.1007/s00429-013-0640-5

**Published:** 2013-10-22

**Authors:** Bing Liu, Lingzhong Fan, Yue Cui, Xiaolong Zhang, Bing Hou, Yonghui Li, Wen Qin, Dawei Wang, Chunshui Yu, Tianzi Jiang

**Affiliations:** 1 Brainnetome Center, Institute of Automation, Chinese Academy of Sciences, Beijing, 100190 China; 2 National Laboratory of Pattern Recognition, Institute of Automation, Chinese Academy of Sciences, Beijing, 100190 China; 3 Queensland Brain Institute, The University of Queensland, Brisbane, QLD 4072 Australia; 4 Department of Radiology, Tianjin Medical University General Hospital, No. 154, Anshan Road, Heping District, Tianjin, 300052 China; 5 Key Laboratory for NeuroInformation of Ministry of Education, School of Life Science and Technology, University of Electronic Science and Technology of China, Chengdu, 610054 China

**Keywords:** *DISC1*, Functional connectivity density, Anatomical connectivity, Thalamus, Thalamic-prefrontal circuit

## Abstract

The Disrupted-in-Schizophrenia 1 (*DISC1*) gene has been thought as a putative susceptibility gene for various psychiatric disorders, and *DISC1* Ser704Cys is associated with variations of brain morphology and function. Moreover, our recent diffusion magnetic resonance imaging (dMRI) study reported that *DISC1* Ser704Cys was associated with information transfer efficiency in the brain anatomical network. However, the effects of the *DISC1* gene on functional brain connectivity and networks, especially for thalamic-prefrontal circuit, which are disrupted in various psychiatric disorders, are largely unknown. Using a functional connectivity density (FCD) mapping method based on functional magnetic resonance imaging data in a large sample of healthy Han Chinese subjects, we first investigated the association between *DISC1* Ser704Cys and short- and long-range FCD hubs. Compared with Ser homozygotes, Cys-allele individuals had increased long-range FCD hubs in the bilateral thalami. The functional and anatomical connectivity of the thalamus to the prefrontal cortex was further analyzed. Significantly increased thalamic-prefrontal functional connectivity and decreased thalamic-prefrontal anatomical connectivity were found in *DISC1* Cys-allele carriers. Our findings provide consistent evidence that the *DISC1* Ser704Cys polymorphism influences the thalamic-prefrontal circuits in humans and may provide new insights into the neural mechanisms that link *DISC1* and the risk for psychiatric disorders.

**Electronic supplementary material:**

The online version of this article (doi:10.1007/s00429-013-0640-5) contains supplementary material, which is available to authorized users.

## Introduction

The Disrupted-in-Schizophrenia 1 (*DISC1*) gene (Millar et al. 2000) first identified in a large Scotland pedigree study (Blackwood et al. 2001) is well established as a genetic risk factor for multiple psychiatric disorders (Chubb et al. 2008; Bradshaw and Porteous 2012). Studies in cell and animal models suggest that DISC1 is functionally involved in several key processes that regulate neural development and brain maturation (Schurov et al. 2004; Clapcote et al. 2007; Duan et al. 2007; Kim et al. 2009; Ming and Song 2009; Brandon and Sawa 2011; Johnstone et al. 2011; Tomita et al. 2011). One common missense variant in the DISC1 gene, Ser704Cys (rs821616), has been independently reported by several groups as associated with an increased risk for various psychiatric disorders (Hashimoto et al. 2006; DeRosse et al. 2007; Palo et al. 2007; Qu et al. 2007; Kim et al. 2008), although a meta-analysis has recently questioned this claim (Mathieson et al. 2012). The associations between this genetic variant and changes in brain morphology and function have been reported (Callicott et al. 2005; Hashimoto et al. 2006; Di Giorgio et al. 2008; Prata et al. 2008; Takahashi et al. 2009; Johnstone et al. 2011; Raznahan et al. 2011; Sprooten et al. 2011; Trost et al. 2013). Moreover, our recent diffusion magnetic resonance imaging (dMRI) study reported that *DISC1* Ser704Cys is associated with information transfer efficiency in the brain anatomical network of healthy subjects (Li et al. 2013). A relevant next question was whether the functional connectivity of the brain, especially in specific circuits, was measurably influenced by the *DISC1* Ser704Cys polymorphism.

The effects of the *DISC1* gene on functional brain connectivity and networks, which have been shown to be disrupted in various psychiatric disorders (Hulvershorn et al. 2011; Rosazza and Minati 2011), are largely unknown. Resting-state functional connectivity, which is measured with functional magnetic resonance imaging (fMRI), can be used to explore the overall organization of functional brain network (van den Heuvel and Hulshoff Pol 2010). Recently, Tomasi and Volkow (2010a, b) proposed a functional connectivity density mapping (FCDM) method to characterize the voxel-wise short- and long-range functional connectivity density (FCD) hubs in the human brain. This data-driven method can effectively calculate individual functional connectivity maps with voxel-wise spatial resolution and overcome the limitations of seed-based methods for identifying and locating functional connectivity hubs in the brain. Here we aimed to use this data-driven method based on resting state fMRI data to investigate the effects of the psychiatric risk variant (*DISC1* Ser704Cys) on the functional connectivity hubs of brain networks and to reveal the functional connectivity changes of the underlying neural circuits in a large sample of healthy Han Chinese subjects. Our hypothesis was that this analytical approach to brain imaging for functional connectivity, especially in the thalamic-prefrontal circuit, might provide new insights into the neural mechanisms that link DISC1 and the risk for psychiatric disorders.

The goals of the present study were as follows: (1) delineate the effects of *DISC1* Ser704Cys on short- and long-range functional connectivity hubs throughout the whole brain; and (2) determine specific functional connectivity circuits that are affected by *DISC1* genetic variants. We first used FCDM based on resting-state fMRI to identify the effects of *DISC1* Ser704Cys on short- and long-range functional connectivity hubs. We hypothesized that the FCD hubs of the thalamus were influenced by *DISC1* Ser704Cys. The FCD hubs that showed significant differences between groups of individuals bearing different *DISC1* genotypes served as the region of interest (ROI) in the following analyses. We next analyzed the corresponding changes of functional and anatomical connectivity of the ROI. Specifically, functional connectivity mapped the correlations between the ROI and all other voxels in the brain, and anatomical connectivity projected the structural pathways of the ROI throughout the whole brain. Accordingly, we established the impact of *DISC1* Ser704Cys on functional and anatomical connectivity of the ROI.

## Materials and methods

### Subjects

We recruited 323 young healthy Han Chinese subjects (157 males and 166 females, mean age = 22.7 years, range 18–31 years) as described in our previous studies (Li et al. 2013). After excluding subjects with missing *DISC1* genotype information and subjects without sufficient neuroimaging data, finally 284 subjects were included in the fMRI analyses and 278 subjects were included in the dMRI analyses (Supplementary Figure 1).

### DISC1 genotyping

We extracted genomic DNA from whole blood using the EZgeneTM Blood gDNA Miniprep Kit (BIOMIGA Inc, San Diego, CA, USA). The *DISC1* Ser704Cys (rs821616) was then genotyped using the PCR and Ligation Detection Reaction (LDR) method as previously described (Li et al. 2013). Nine subjects with missing genotype data were excluded for further analysis. The distribution of *DISC1* genotypes in our samples (SerSer: *N* = 218, SerCys: *N* = 86, CysCys: *N* = 10) did not significantly deviate from the Hardy–Weinberg equilibrium (*χ* ^2^ = 0.18,* df *= 1, *P* = 0.67).

### Image data acquisition

All subjects were scanned on the same Signa HDx 3.0 T magnetic resonance scanner (GE Healthcare; Milwaukee, WI, USA). During scanning, we used foam padding and ear plugs to reduce head motion and scanning noise, respectively. All the participants underwent a high-resolution T1-weighted brain volume (BRAVO) 3D MRI sequence [repetition time (TR) = 8.1 ms; echo time (TE) = 3.1 ms; flip angle = 13°; voxel size = 1 × 1 × 1 mm^3^; 176 sagittal slices]. Subsequently, resting-state functional imaging was collected with a single-shot gradient-echo echo-planar-imaging (SS-GRE-EPI) sequence sensitive to blood oxygenation level-dependent (BOLD) contrast [TR = 2,000 ms; TE = 30 ms; field of view (FOV) = 240 × 240 mm^2^; matrix = 64 × 64; flip angle = 90°; voxel size = 3.75 × 3.75 × 4.0 mm^3^; 40 slices and 180 volumes]. During resting-state fMRI, all subjects were instructed to be eye-closed and to move as little as possible without falling asleep. Finally, the diffusion-weighted images were collected using echo-planar imaging with 45 contiguous 3 mm axial slices and 55 non-collinear diffusion gradients together with three non-diffusion-weighted acquisitions (TR = 10,000 ms; TE = 64.2 ms;* b*-value = 1,000 s/mm^2^; matrix = 128 × 128; FOV = 256 × 256 mm^2^; flip angle = 90°; voxel size = 2 × 2 × 3 mm^3^).

### fMRI preprocessing

Fourteen subjects were excluded for apparent inter-slice motion artifacts of their raw fMRI data as evaluated by the visual inspection of two radiologists who were blinded to genotype (Yu and Qin). Further data preprocessing was carried out in the 300 remaining subjects using FSL tools (www.fmrib.ox.ac.uk/fsl) and in-house programs. A series of preprocessing steps were performed: (1) discarding the first ten volumes for the magnetization equilibrium; (2) slice timing correction; (3) head motion correction; (4) non-brain removal; (5) temporal filtering (0.01–0.1 Hz); and (6) regressing nuisance signals (six motion parameters). The registration of the resting-state data to high-resolution T1-weighted images and the T1 data to the 3-mm isotropic MNI-152 standard space template (Montreal Neurological Institute) were carried out. The resulting transformation matrices were combined to obtain a native MNI space transformation matrix and its inverse. Sixteen subjects who had a maximum displacement in any of the cardinal directions (*x*, *y*, *z*) larger than 2 mm or a maximum spin (*x*, *y*, *z*) larger than 2° were excluded from subsequent analysis. In total, 284 subjects were included in the fMRI data analysis (Supplementary Figure 1). The distribution of the DISC1 genotype in the 284 remaining subjects (SerSer: *N* = 197, SerCys: *N* = 79, CysCys: *N* = 8) still did not significantly deviate from the Hardy–Weinberg equilibrium (*χ* ^2^ = 0.001,* df *= 1, *P* = 0.98). Moreover, there was no significant bias in the genotype distrubition of the 30 exclusionary (SerSer: *N* = 21, SerCys: *N* = 7, CysCys: *N* = 2) and the 284 remaining samples (*χ* ^2^ = 1.20,* df *= 2, *P* = 0.55).

### Short- and long-range FCD

Here, we used FCDM to evaluate the individual FCD maps with voxel-vise spatial resolution (Tomasi and Volkow 2010a, 2011). First, we computed the global FCD based on prior knowledge that two voxels are considered functionally connected if their Pearson correlation coefficient is greater than 0.6 (Tomasi and Volkow 2011). The global FCD of a given voxel was defined as the number of functional connections between this voxel and all other voxels in the brain. Second, we obtained the short-range FCD using a “growing” algorithm. In this algorithm, given a voxel *x*_0_, an additional voxel *x*_*j*_, was added to the list of neighbors of *x*_0_ if it was adjacent to a voxel that was linked to *x*_0_ by a continuous path of functionally connected voxels and the correlation coefficient between *x*_0_ and *x*_*j*_ was >0.6. This calculation was repeated for all voxels that were adjacent to the neighbors of *x*_0_ in an iterative manner until no new neighbors could be added to the list. The short-range FCD of *x*_0_ was defined as the number of elements in the list of neighbors. The strength of the long-range FCD was equated to the difference between the global FCD and the short-range FCD. These calculations were performed on all voxels in the brain. Therefore, the strength of the short-range FCD of a given voxel reflects the functional correlation between that voxel and other voxels within a local cluster, and the strength of the long-range FCD reflects the functional correlation between a given voxel and other voxels located at a distance. Finally, the short and long-range FCD maps were spatially smoothed using a Gaussian kernel of full-width at half-maximum (FWHM) of 8 mm. The short and long-range FCD of each voxel was divided by the mean value of each individual to normalize the distribution of the FCD strength.

### Functional connectivity analyses

We calculated the voxel-wise functional connectivity throughout the entire brain by taking the brain regions that revealed different FCD hubs between *DISC1* Ser704Cys genotypes as the ROI. The functional connectivity map of each individual was computed by averaging the BOLD time series in the ROI and then computing the Pearson’s correlation coefficient between the seed average time series and those from each voxel in the brain. The resulting correlations were transformed to approximate a Gaussian distribution using Fisher’s *z* transformation. In this way, a functional connectivity map of the ROI for each participant was produced.

### dMRI preprocessing and anatomical connectivity analyses

Two radiologists (Yu and Qin) visually inspected the raw dMRI data for apparent artifacts arising from subject motion and instrument malfunction. After excluding 36 subjects (SerSer: *N* = 20; SerCys: *N* = 14; CysCys: *N* = 2), the 278 remaining subjects, including 198 Ser homozygotes and 80 Cys-allele carriers (SerCys: *N* = 72; CysCys: *N* = 8), underwent further dMRI data analysis (Supplementary Figure 1). There was no significant bias in the genotype distribution of the 36 exclusionary and 278 remaining samples (*χ* ^2^ = 3.57,* df *= 2, *P* = 0.17). The distribution of the DISC1 genotype in the 278 remaining subjects still did not significantly deviate from the Hardy–Weinberg equilibrium (*χ* ^2^ = 0.22,* df *= 1, *P* = 0.63).

We further described the anatomical connectivity map of the ROI with different FCD hubs and aimed to find whether specific anatomical connectivity contributed to the *DISC1* genetic effects. First, the significant ROIs obtained in the previous FCD analyses were transformed into the individual diffusion space. For this purpose, the skull-stripped T1-weighted images of each subject were co-registered with the non-diffusion-weighted images (*b* = 0 s/mm^2^) using FLIRT. Then, the T1 images in diffusion space were wrapped linearly and nonlinearly into the MNI (Montreal Neurological Institute) space using FLIRT and FNIRT. After that, we applied the inverse linear and non-linear transformations to transform the ROIs into the individual diffusion space. Second, the diffusion MRI data were processed using the FMRIB Software Library (FSL). Eddy-current distortions and simple head motion were corrected by applying affine registration of all volumes to a target volume with no diffusion weighting, and three-dimensional maps of the diffusion tensor and the fractional anisotropy (FA) values were calculated using FSL. Moreover, the local probability distributions of fiber directions were estimated at each voxel (Behrens et al. 2003). We chose the computation model and allowed for the automatic estimation of two fiber directions within each voxel (Behrens et al. 2007). Then, we drew 5,000 samples from the connectivity distribution for each voxel in the ROI and calculated the connection probability of each voxel. To reduce the false-positive rates, the fiber tracking results were thresholded to exclude all connection probability values of *P* < 0.002 (10 out of 5,000 samples) (Wang et al. 2012; Zhang et al. 2012). Next, the probability connectivity map for each subject was binarized with *P* and transformed into standard MNI space. A population-based anatomical connectivity map of the ROI with different FCD hubs was obtained by averaging the transformed individual binary maps. Finally, we binarized the population-based anatomical connectivity map with a threshold of 80 % (the threshold was tested from 50 to 100 % in all subjects with no differences on the following analysis) as a mask for the following voxel-based FA analysis between *DISC1* Cys-allele carriers and Ser homozygotes.

### Statistical analyses

We used a Pearson Chi square test to test for differences in gender and a two-sample *t* test to test for differences in age, educational year, and intelligence quotient (IQ) score between the two genotype groups. Voxel-wise two-sample *t*-tests with four covariates (age, gender, educational year, and IQ score) were implemented in SPM8 to map group differences in the strength of short- and long-range FCDs, functional and anatomical connectivity from the ROI between *DISC1* Cys-allele carriers and Ser homozygotes. For all imaging analyses, the Monto Carlo simulation method was used throughout the entire brain to correct for multiple comparisons by AlphaSim program (corrected *P* < 0.01). For the thalamic functional connectivity analyses, corrected for multiple comparisons was used within prefrontal cortex as defined in previous studies (Wei et al. 2012).

## Results

### Demographic information

In total, 323 young subjects were recruited. Of these, 284 subjects were included in the fMRI data analyses and 278 were included in the previous and current dMRI data analyses (Table 1; Supplementary Figure 1). All of the individuals in the study were categorized into two groups based on *DISC1* Ser704Cys genotypes: Ser homozygotes and Cys-allele carriers. No significant differences were found in age, gender, educational year, and IQ score between two groups (Table 1).

**Table 1:** Demographic information

	Subjects included in fMRI data analyses			Subjects included in dMRI data analyses		
	SerSer (*n* = 197)	Cys-allele (*n* = 87)	*P*-value	SerSer (*n* = 198)	Cys-allele (*n* = 80)	*P*-value
Gender	93 M/104 F	36 M/51 F	0.37	94 M/104 F	32 M/48 F	0.26
Age in years*	22.75 (2.53)	22.83 (2.24)	0.81	22.77 (2.50)	23.09 (2.19)	0.33
Education in years*	15.56 (2.51)	15.49 (2.72)	0.84	15.61 (2.51)	15.54 (3.03)	0.85
FSIQ*	117.02 (9.59)	117.38 (7.71)	0.75	116.84 (9.82)	117.41 (8.14)	0.65

*F* female, *M* male, *FSIQ* full scale intelligence quotient

* Values are mean (SD)

### Functional connectivity density analysis

**Fig. 1:**
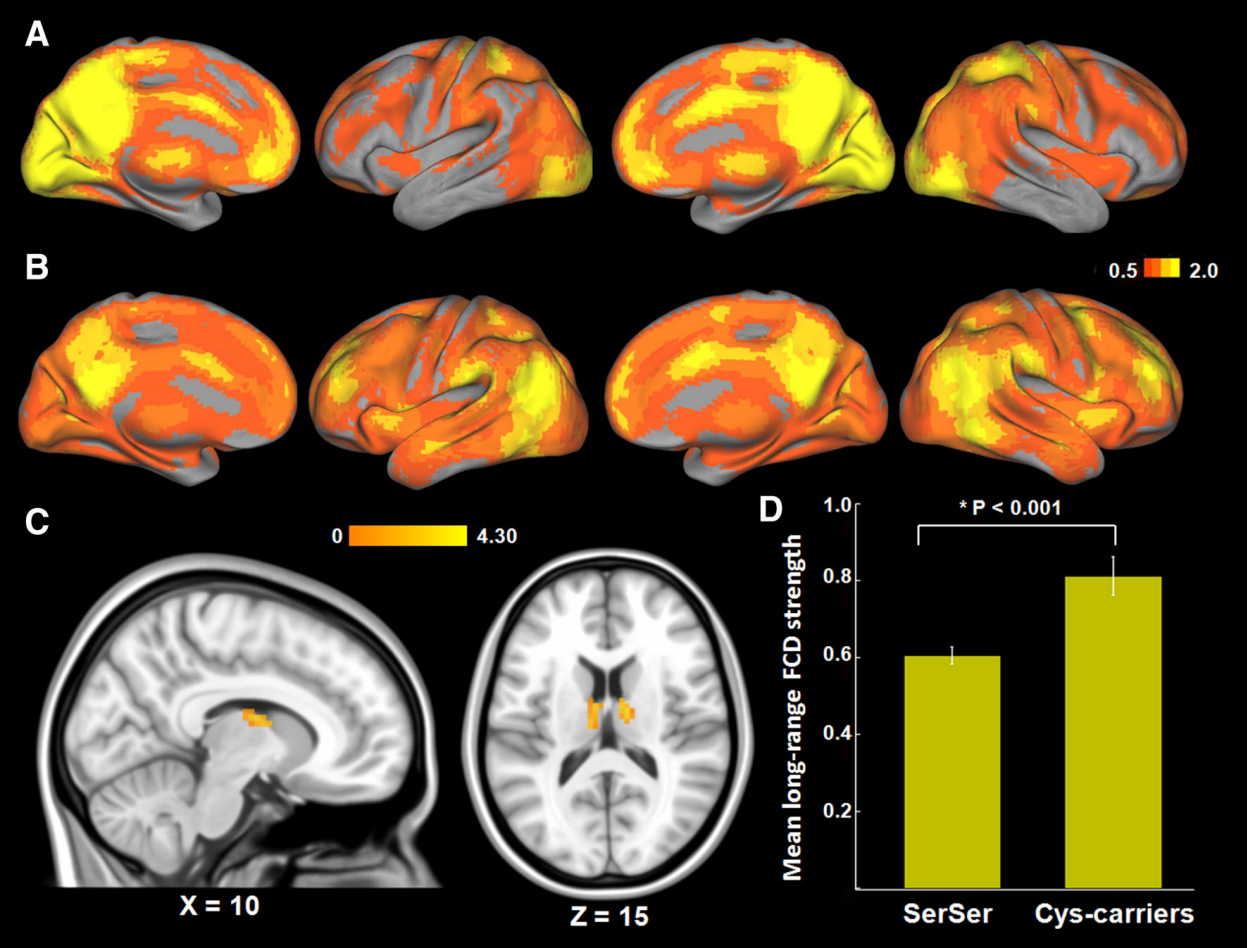
Results of FCD analyses. **a**, **b** Shows the spatial distribution of the average short-range FCD and long-range FCD, respectively, superimposed on the cerebral cortex for all subjects. **c** Significantly increased long-range FCD of Cys-allele carriers compared with Ser homozygotes (corrected *P* < 0.01). **d** For illustrative purposes, the average value of the long-range FCD of bilateral thalami (within significant clusters) was displayed in the *bar graph* for two different genotype groups (mean ± SEM)

We calculated the short- and long-range FCD maps for each individual, and group comparisons were performed. Using the averaged short- and long-range FCD maps (Fig. 1a, b), we detected similar patterns as observed in previous studies (Tomasi and Volkow 2010a, 2011). The short- and long-range FCD patterns were observed bilaterally, with maximal magnitude in posterior cingulate, occipital and prefrontal cortices. Compared with Ser homozygotes, Cys-allele carriers showed significantly increased long-range FCD in the bilateral thalami (peak voxel MNI coordinate: *x* = 12, *y* = −9, *z* = 15; *T* = 4.29; cluster size = 67), mainly located at the mediodorsal thalamic nucleus (Fig. 1c, d). No significant differences in short-range FCD were found between the two *DISC1* genotypes.

### Thalamic functional connectivity analysis

**Fig. 2:**
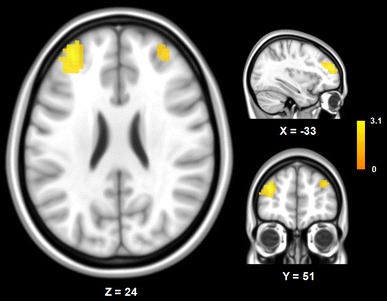
Thalamic functional connectivity. The increased functional connectivity of the thalamus in Cys-allele carriers was found in the dorsolateral prefrontal cortex compared with Ser homozygotes (corrected *P* < 0.01, corrected within the prefrontal region)

Since the long-range FCD in the bilateral thalami has been found to be significantly increased Cys-allele carriers compared with Ser homozygotes as we hypothesized, we further investigated the specific functional connectivity circuits that are affected by *DISC1* genetic variants by ROI-based functional connectivity analyses. To determine the effects of DISC1 Ser704Cys on functional connectivity to the thalamus, we performed voxel-vise functional connectivity analyses using the thalamic region that showed significantly different long-range FCD as the ROI. We found that Cys-allele carriers showed increased thalamic functional connectivity in the bilateral dorsolateral prefrontal cortex (DLPFC) than did the Ser homozygotes (Fig. 2; Supplementary Table 1).

### Thalamic anatomical connectivity analysis

**Fig. 3:**
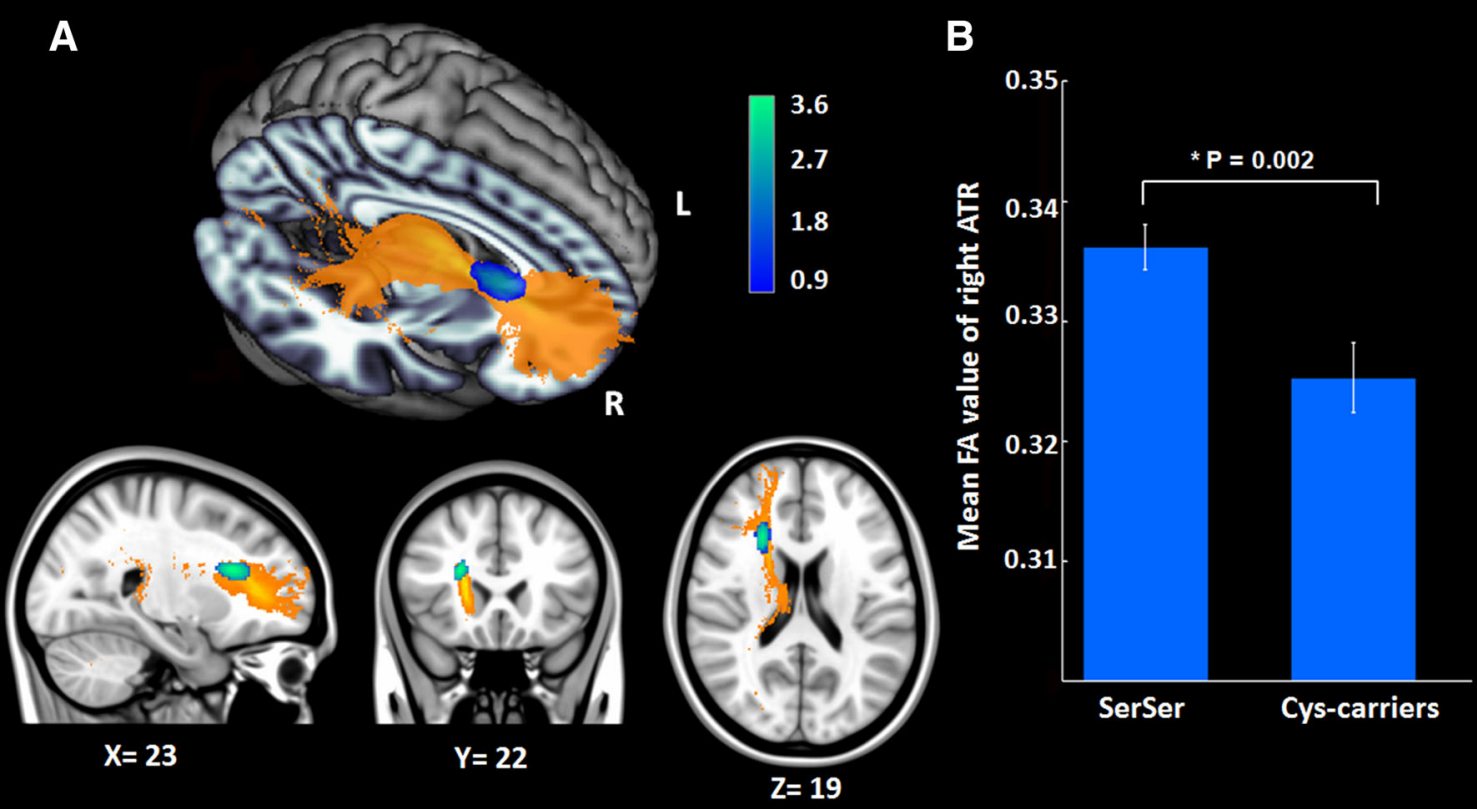
Thalamic anatomical connectivity. **a** The significantly decreased FA of Cys-allele carriers compared to Ser homozygotes was superimposed on the tract of the anterior thalamic radiation (corrected *P* < 0.01). **b** For illustrative purposes, the average value of the thalamic-prefrontal anatomical connectivity (within significant clusters) was displayed in the *bar graph* for two different genotype groups (mean ± SEM)

Using probabilistic tractography of dMRI, we reconstructed thalamic pathways throughout the whole brain for each individual. Resulting fibers were registered onto the standard MNI space (Supplementary Figure 2) and submitted to a voxel-wise statistical analysis. With further investigation, we found that, compared with *DISC1* Ser homozygous individuals, Cys-allele carriers showed significantly decreased FA values in the right anterior thalamic radiation (ATR) (peak voxel MNI coordinate: *x* = 24, *y* = 22, *z* = 20; *T* = 3.69; cluster size = 76) (Fig. 3), which carries major nerve fibers between the thalamus and the prefrontal cortex.

## Discussion

Using data-driven methods based on fMRI data in a large sample of healthy Han Chinese subjects, our findings have provided consistent evidence that the *DISC1* Ser704Cys polymorphism influences the thalamic-prefrontal circuits in humans. Based on resting-state fMRI data, we first investigated the association between *DISC1* Ser704Cys and short- and long-range functional connectivity hubs using FCDM. Compared with Ser homozygotes, Cys-allele individuals had increased long-range FCD hubs in the bilateral thalami. The functional and anatomical connectivity of the thalamus to the prefrontal cortex was further analyzed. Significantly increased thalamic-prefrontal functional connectivity and significantly decreased thalamic-prefrontal anatomical connectivity were found in *DISC1* Cys-allele carriers when compared to Ser homozygotes.

One of the major findings of the present study was the effect of *DISC1* Ser704Cys on the long-range FCD in the bilateral thalami, especially on the mediodorsal nucleus of the thalamus. Ever since the *DISC1* gene was originally discovered in a Scottish pedigree, the accumulating biological evidence has indicated that DISC1 plays diverse and important roles in brain development and maturation, including early neurodevelopment, synaptic regulation, and oligodendrocyte development (Brandon et al. 2009; Brandon and Sawa 2011; Johnstone et al. 2011). These roles are consistent with the current hypotheses for the etiopathology of major psychiatric disorders. *DISC1* Ser704Cys is one of the vital non-synonymous mutations of the *DISC1* gene, and it has been widely reported to be associated with different clinical and structural brain phenotypes both in patients and healthy individuals, though the results are inconsistent (Callicott et al. 2005; Hashimoto et al. 2006; DeRosse et al. 2007; Qu et al. 2007; Di Giorgio et al. 2008; Sprooten et al. 2011). In particular, the *DISC1* Cys allele was positively associated with schizophrenia in a study of Han Chinese subjects (Qu et al. 2007). Individuals carrying the Cys allele for Ser704Cys have reduced gray matter volume in the cingulate cortex and decreased FA in prefrontal white matter (Hashimoto et al. 2006). Moreover, evidence indicates that the Ser704Cys genetic variant may inhibit the migration of neurons in the developing neocortex (Singh et al. 2011) and alter oligomeric assembly (Narayanan et al. 2011). On the other hand, the thalamus has been universally believed to serve as a relay of information processing with its nerve fibers projecting to the cerebral cortex in all directions with extensively interconnected major cortical circuits (Andreasen 1997; Briggs and Usrey 2008). Abnormal structure and function of the thalamus as observed in postmortem and neuroimaging studies, respectively, has been widely reported in various psychiatric disorders (Andreasen 1997; Byne et al. 2009; Adriano et al. 2010; Cronenwett and Csernansky 2010). As a psychiatric risk gene, *DISC1* would, therefore, be expected to affect thalamic function. We speculate that the increased long-range FCD observed in bilateral thalami in this study may be a manifestation of the compensatory effect of thalamic connectivity because of the weaker information processing function of the thalamus in *DISC1* Cys-allele carriers compared with Ser homozygotes, with more thalamic functional connectivity required to achieve the same behavioral output.

The most significant differences of long-range FCD are observed in the mediodorsal nucleus of the thalamus, which mainly provides projections to and from various regions of the prefrontal cortex (Andreasen 1997). Previous anatomical and functional neuroimaging findings have shown disrupted prefrontal-thalamic circuitry in schizophrenia (Lewis 2000; Lehrer et al. 2005; Rose et al. 2006; Pakkenberg et al. 2009; Marenco et al. 2012; Woodward et al. 2012) and other psychiatric disorders (Strakowski et al. 2005). Alterations in thalamic-prefrontal circuitry might be a pivotal endophenotypic trait that should be studied to understand the neural mechanisms of genes associated with psychiatric disorders. Using functional connectivity analyses of resting-state fMRI, we calculated the thalamic functional connectivity map for each individual and performed voxel-wise comparisons between the two genotypes. *DISC1* Cys-allele individuals showed increased thalamic-prefrontal functional connectivity compared with Ser homozygotes. Here we further used two complementary approaches based on diffusion MRI (dMRI), probabilistic fiber tractography and voxel-wise morphometry of FA, to “dissect” the affected white matter pathways from the thalamus and quantify the altered white-matter integrity on an individual basis (Mori and Zhang 2006; Behrens et al. 2007). We reconstructed thalamic pathways throughout the whole brain and further performed a voxel-wise statistical analysis comparing different *DISC1* genotypes. Individuals with the *DISC1* Cys-allele exhibited less thalamic-prefrontal anatomical connectivity, particularly involving the right ATR, which is one of the most important fibers connecting the mediodorsal thalamic nucleus and the prefrontal cortex. The finding of current voxel-wise analyses of the thalamic white matter pathways was consistent with our tract-based analyses of the previous study, which has reported that of the 20 major fiber tracts only the white matter integrity of the ATR has the trend to differ between the individuals with different *DISC1* Ser704Cys genotypes (Li et al. 2013). The white matter integrity of the ATR has been widely reported to be decreased in psychiatric disorders (McIntosh et al. 2008; Mamah et al. 2010; Perez-Iglesias et al. 2010) and in psychosis-risk-associated neuregulin-1 variants (Sprooten et al. 2009; Barnes et al. 2012). These findings combined with our previous study consistently support that the *DISC1* Ser704Cys polymorphism, as a key psychosis-risk-related genetic variation, impacts the thalamic-prefrontal functional and anatomical connectivity. These effects provide a possible explanation for the abnormal thalamic function and disrupted thalamic-prefrontal circuit that is often reported in psychiatric disorders. These effects also suggest that there is a potential mechanistic link between *DISC1* Ser704Cys and the risk for psychiatric disorders.

Using resting-state fMRI and dMRI data, our present study combining with the previous finding reported that *DISC1* Cys-allele carriers had significantly increased long-range FCD in the thalamus, increased thalamic-prefrontal functional connectivity, and decreased thalamic-prefrontal anatomical connectivity compared with Ser homozygotes in a large sample of healthy subjects. Although some of the functional connectivity may have an anatomical basis, the relationship between functional and anatomical connectivity is complex. For example, anatomical connectivity measured by dMRI is nearly uniformly decreased in schizophrenia (Ellison-Wright and Bullmore 2009; Bora et al. 2011). On the other hand, the founding of functional connectivity in schizophrenia is inconsistent regardless of the specific connections or the directions (Skudlarski et al. 2010; Fornito et al. 2012). Our findings of increased functional connectivity may represent increased neural effort due to decreased anatomical connectivity in the thalamic-prefrontal circuit. This type of compensatory mechanism may limit the effects of genes related to psychiatric disorders and help maintain a normal range of behavioral performance in healthy subjects. The compensatory mechanism of increased thalamic-prefrontal functional connectivity in healthy subjects may conversely explain the finding of reduced prefrontal-thalamic functional connectivity in schizophrenia (Woodward et al. 2012). However, more biological evidences are needed to elucidate the relationship between anatomical and functional connectivity.

We performed comprehensive functional connectivity analyses based on brain imaging data of healthy individuals with different *DISC1* genotypes, therefore providing new insights into the neural mechanisms that link *DISC1* Ser704Cys and risk for psychiatric disorders by the imaging endophenotype of thalamic-prefrontal circuit. Here it should be noted that another important functional polymorphism of *DISC1*, Leu607Phe, was not considered in the present study because the sample size carrying the minor allele in all the subjects (3/323) is too few to make group comparisons. Moreover, there are still large gaps between the genetic variants and neural connectivity. Systematic studies using animal models or other techniques may provide more solid biological evidence to explain this relationship.

## Electronic supplementary material

Below is the link to the electronic supplementary material.

Supplementary material 1 (DOC 402 kb)
