# Supplementary material for: DISC1 Ser704Cys impacts thalamic-prefrontal connectivity
Source: Brain Struct Funct. 2013 Oct 22;220(1):91–100. doi: 10.1007/s00429-013-0640-5 (PMC4286634; doi:10.1007/s00429-013-0640-5)
Supplement: Supplementary file 1 — Supplementary material 1 (DOC 402 kb) [file 429_2013_640_MOESM1_ESM.doc]

***DISC1* Ser704Cys Impacts Thalamic-Prefrontal Connectivity**

Bing Liu^1,2#^, Lingzhong Fan^1,#^, Yue Cui^1,2^, Xiaolong Zhang^1^, Bing Hou^1,2^, Yonghui Li^4^, Wen Qin^3^, Dawei Wang^3^, Chunshui Yu^3, *^ , Tianzi Jiang^1, 2, 4, 5, *^

^1^ Brainnetome Center, ^2^ National Laboratory of Pattern Recognition, Institute of Automation, Chinese Academy of Sciences, Beijing 100190, China

^3^ Department of Radiology, Tianjin Medical University General Hospital, Tianjin 300052, China

^4^ Queensland Brain Institute, The University of Queensland, Brisbane, QLD 4072, Australia

^5^ Key Laboratory for NeuroInformation of Ministry of Education, School of Life Science and Technology, University of Electronic Science and Technology of China, Chengdu 610054, China

# Bing Liu and Lingzhong Fan contributed equally to this work and should be considered co-first authors.

**Supplementary Figure 1**. Flowchart of subjects included in the fMRI/dMRI data analyses.

**323 subjects**

**314 subjects**

**300 subjects**

**278 subjects**

**9 excluded for failure of genotyping**

**14 excluded for serious artifacts of their fMRI data**

**36 excluded for serious artifacts of their dMRI data**

**284 subjects**

**16 excluded for their maximum displacements in x, y or z directions >2mm, or maximum rotations around x ~ z axes >2°**

**fMRI analysis**

**dMRI analysis**

**Supplementary Figure 2**. The averaged probability connectivity maps of the thalamus to the whole brain for all individuals in the MNI space.


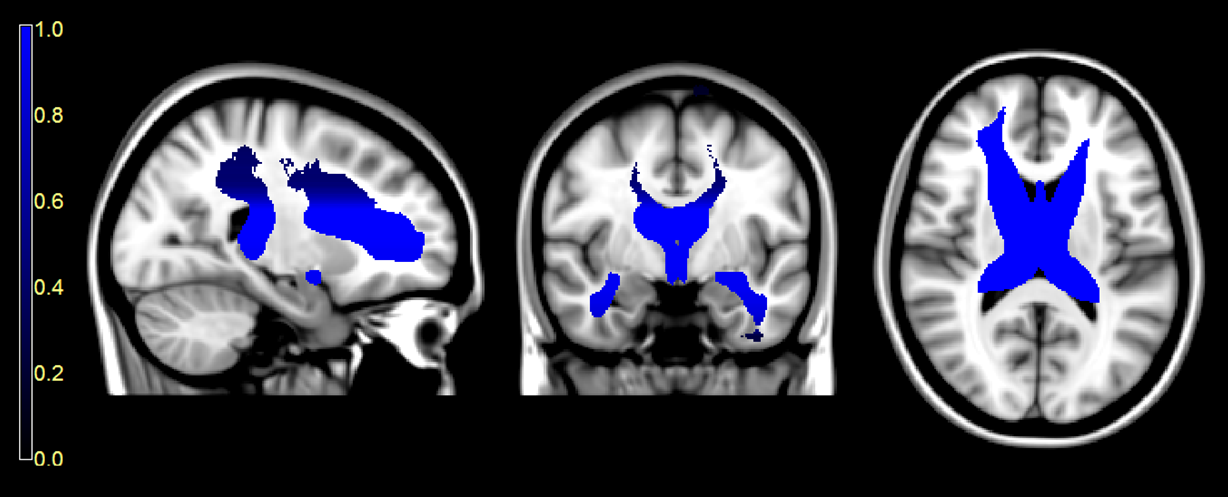


**Supplementary Table 1**. Regions of group differences in thalamic functional connectivity analyses (Cys-allele carriers > Ser homozygotes, corrected within the prefrontal region).

| **Region** | **Brodmann area** | **Cluster size** | **T-value** | **Peak coordinate** |
| --- | --- | --- | --- | --- |
| Left middle frontal gyrus | 10 | 168 | 3.10 | -33 51 24 |
| Right middle frontal gyrus | 10 | 34 | 2.51 | 33 51 27 |
